# GenAI-supported project-based product design learning: differential changes in multidimensional learning outcomes

**DOI:** 10.3389/fpsyg.2026.1962323

**Published:** 2026-10-07

**Authors:** Yizhou Chen, Yingqi Zhao, Kenny S. L. Cheah, Nur Yuhanis Mohd Nasir

**Affiliations:** 1 Institute of Advanced Studies, University of Malaya, Kuala Lumpur, Malaysia; 2 Faculty of Social Science and Liberal Art, UCSI University, Cheras, Malaysia; 3 Department of Education Management, Planning and Policy, Faculty of Education, Universiti Malaya, Kuala Lumpur, Malaysia; 4 Department of Malay Language, Faculty of Modern Languages and Communication, Universiti Putra Malaysia, Serdang, Malaysia

**Keywords:** cognitive learning, generative artificial intelligence, learning outcome, product design education, project-based learning (PjBL)

## Abstract

Generative artificial intelligence (GenAI) is increasingly used in design education, yet evidence remains limited on whether its educational value derives from tool access itself or from how human–AI interaction is structured within project work. This quasi-experimental pre-test–post-test study compared a 4-week GenAI-Integrated Project-Based Learning Module (GIPLM) with traditional project-based learning (PjBL) in an undergraduate product design course (*N* = 60). Rather than treating GenAI as a stand-alone content-generation tool, GIPLM embedded GenAI as process scaffolding across four stages: product and requirement analysis, user research and knowledge structuring, ideation with multimodal visualization and 3D prototyping, and feedback, validation, and reflective presentation. Learning outcomes were assessed across cognitive, affective, and psychomotor-related domains, together with course performance. According to a mixed-design ANOVA, the GenAI-supported group was associated with significantly greater gains than the control group in cognitive understanding, problem-solving and creativity, fundamental tool operation, advanced design application, and final academic performance. Because advanced design application differed at baseline, ANCOVA was additionally conducted; the post-test group effect remained significant after controlling for pre-test scores, *F*_(1, 57)_ = 20.519, *p* < 0.001, partial η^2^ = 0.265. Engagement and participation and ethical and user-centered responsibility improved over time but showed no significant Time × Group interactions. These findings suggest that the observed advantages are more consistent with structured, workflow-embedded GenAI scaffolding than with AI access alone, with stronger short-term effects on cognitive, procedural, and performance outcomes than on affective development.

## 1 Introduction

Open-ended project work requires learners to coordinate prior knowledge, problem representation, feedback, and iterative decision-making under conditions of uncertainty (Beneroso and Robinson, 2022; Liu et al., 2026). This issue is particularly relevant in product design education, where students move repeatedly among problem framing, user research, concept generation, visual representation, modeling, and evaluation (Miska et al., 2022; Saadi and Yang, 2023). These activities require students not only to acquire disciplinary knowledge but also to organize information, generate alternatives, justify decisions, and translate ideas across multiple representational forms (Tsai and Hsu, 2024; Zakaria and Lim, 2022). For novice learners, the coordination of these activities can be demanding because the problem is often ambiguous and the criteria for acceptable solutions are not fully specified in advance. A central challenge, therefore, is to understand how instructional support influences the cognitive demands and learning opportunities embedded in such tasks.

Project-based learning (PjBL) is widely implemented in product design education because its emphasis on authentic problems, sustained inquiry, collaboration, and iterative refinement mirrors professional design practice (Hu and Chu, 2022; Borgianni et al., 2025; Shashidhara et al., 2026). Engaging students in authentic design projects facilitates both knowledge construction and professional skill development (Brundiers and Wiek, 2013; Zhang and Ma, 2023). However, the open-ended nature of PjBL may create substantial cognitive and regulatory demands. Students are expected to identify relevant information, compare competing alternatives, integrate feedback, and revise intermediate representations while maintaining a coherent understanding of the design problem. When support is delayed or insufficient, these demands may contribute to cognitive overload, pre-mature solution selection, or uneven participation in iterative learning processes (Kokotsaki et al., 2016; Sukacke et al., 2022).

The rapid development of generative artificial intelligence (GenAI) offers new opportunities to support PjBL. Rather than serving solely as a productivity tool, GenAI may function as embedded cognitive scaffolding. It can provide structured prompts, alternative representations, and immediate feedback, potentially reducing ambiguity during concept development (Schmitt-Fumian et al., 2025). Because its feedback is not mediated by interpersonal judgment, GenAI may also reduce evaluation anxiety and encourage students to articulate early-stage ideas (Zhang and Xu, 2025). These functions are particularly relevant in design education, where ideation and visual representation are central to the learning process. By supporting text generation, sketch creation, and scene rendering, GenAI has the potential to facilitate externalization and sustain iterative design processes (Bartlett and Camba, 2024; Mu, 2024; Ansone et al., 2025). This suggests that GenAI may influence not only task efficiency but also the ways in which cognitive effort, affective engagement, and practical skills are enacted throughout project work.

As interest in GenAI-supported design education grows, the key issue is how GenAI can enhance learning under appropriate pedagogical conditions without undermining the processes through which design competence develops. Previous reviews have shown that GenAI can deliver rapid feedback, facilitate idea generation, and enhance engagement, critical thinking, and problem-solving skills (Qian, 2025; Wang et al., 2025). Studies in design education have likewise reported benefits for conceptual development, visual exploration, and creative support (Abrusci et al., 2025; Medel-Vera et al., 2025). However, a contrasting body of evidence points to a fundamental pedagogical tension. Exposure to AI-generated examples may increase design fixation while reducing the quantity, diversity, and originality of ideas (Wadinambiarachchi et al., 2024). Similarly, although GenAI assistance can improve individual outputs, it may also homogenize collective production (Doshi and Hauser, 2024). Concerns have also been raised that the rapid generation of outputs may weaken students' creative confidence, agency, and sense of ownership when they adopt, rather than critically transform, AI-generated suggestions (Fleischmann, 2025; Habib et al., 2024). These mixed findings suggest that the educational value of GenAI depends less on access to the technology itself than on how human-AI interaction is structured within the learning process. However, existing studies in design education have largely focused on isolated ideation activities, short-term tool use, learner perceptions, or final artifacts. Consequently, there is limited evidence as to whether embedding GenAI as iterative scaffolding throughout authentic PjBL leads to differentiated changes across cognitive, affective, and psychomotor outcomes, or whether such changes translate into formal academic performance relative to traditional PjBL. This process-related, multidimensional uncertainty represents the key research gap examined in this study.

To address these gaps, this study developed a structured instructional module, termed the GenAI-Integrated Project-Based Learning Module (GIPLM), and examined differences associated with this instructional condition using a quasi-experimental design within an undergraduate product design program in China. Students in the experimental group (EG) used selected GenAI tools to support investigation, concept generation, visual exploration, scene rendering, and 3D modeling throughout the project. In contrast, students in the control group (CG) completed the same project through traditional PjBL under instructor guidance, without GenAI integration. Both groups undertook the same 4-week design project, ensuring comparability across instructional conditions. Accordingly, the study compares a workflow-embedded, structured GenAI-supported condition with conventional PjBL, rather than a simple comparison of AI access vs. non-access. The study examines whether this pedagogical configuration is associated with differential changes in multidimensional learning outcomes and academic performance.

The investigation is guided by three interrelated research questions:

(1) Compared with conventional project-based learning, is GenAI-supported project-based learning associated with greater improvements in students' multidimensional learning outcomes?

(2) Which dimensions of learning outcomes show significant differential change between GenAI-supported and conventional PjBL?

(3) Compared with conventional project-based learning, does GenAI-supported project-based learning result in significant differences in students' academic course performance?

This study contributes to research on GenAI-supported learning by examining its integration within an authentic project-based product design context. Rather than treating GenAI primarily as a content-generation tool, the study considers its role as structured support across inquiry, ideation, visualization, evaluation, and revision activities. By examining cognitive, affective, and psychomotor learning outcomes alongside course performance, the study explores whether changes associated with GenAI-supported PjBL are evident across different dimensions of learning. This perspective may help clarify the conditions under which GenAI can complement established project-based learning practices. It also provides a basis for further investigation of how AI-supported scaffolding relates to students' learning processes in open-ended and practice-oriented educational settings.

## 2 Literature review

### 2.1 Multidimensional learning outcomes

Learning outcomes denote what students should know, understand, or execute upon successful completion of a learning process (Gudeva et al., 2012; Mahajan and Singh, 2017; Wagenaar, 2014). The concept signals a transition from input-driven models focused on teaching content and instructional hours, to outcome-oriented frameworks centered on demonstrated competence (Kumpas-Lenk et al., 2018). Bloom's Taxonomy is widely regarded as one of the most influential theoretical frameworks for defining and assessing learning outcomes in educational research (Momen et al., 2023). By classifying learning outcomes into the cognitive, affective and psychomotor domains and emphasizing the hierarchical structure of each domain (Anderson and Krathwohl, 2001; Dave, 1970; Krathwohl et al., 1964), the taxonomy provides a structured foundation for multidimensional assessment in complex learning environments.

In product design education, this three-domain framework aligns closely with the demands of the discipline. The cognitive domain reflects progression from foundational knowledge to higher-order thinking (Anderson and Krathwohl, 2001). Students first develop an understanding of design principles and core concepts (Bodisco and Palmer, 2020; Dillenhöfer et al., 2025), before progressing to application, analysis, evaluation, and ultimately the creative integration of knowledge across domains (Qamar et al., 2016). Creating represents not only the highest level of cognitive development but also a key indicator of successful learning in product design.

The affective domain concerns the development of motivation, attitudes, and professional values (Krathwohl et al., 1964). Through engagement with authentic design problems, students may gradually internalize values such as user-centeredness, sustainability, and social responsibility (Lopez and Bhutto, 2023; Santana and de Deus Lopes, 2024; Hariharasakthisudhan et al., 2025). These studies suggest that affective learning in design extends beyond changes in attitude to encompass the gradual alignment of professional values through situated practice and interaction with stakeholders.

The psychomotor domain encompasses the technical and expressive skills central to design practice, including modeling, prototyping, and digital visualization. Skill development typically progresses from imitation to precision and ultimately to integration (Dave, 1970). For example, a novice design student may initially struggle with the manual dexterity required for foam modeling or the complex interface of professional design software (Lee-Remond et al., 2025). Through repeated practice and feedback, however, the student gradually achieves a level of precision at which measurements are accurate and errors are minimized. Ultimately, at the naturalization stage, the student can execute complex organic surfacing or high-fidelity prototyping with such fluency that the tools become an almost invisible extension of their intent (Wheeler, 2019). As technical execution becomes increasingly automated, students are able to devote greater cognitive attention to ideation and refinement, allowing technical tools to support, rather than constrain, the creative process.

Bloom-related frameworks are used in this study primarily as an organizing structure for learning outcomes rather than as a causal account of GenAI-supported learning. The availability of generated information and representations may alter how learners distribute effort across retrieval, interpretation, evaluation, and creation (Obergassel et al., 2025), but this does not imply that lower-level cognitive processes become unnecessary. Instead, AI-supported environments may change the sequence and visibility of cognitive work and may create a distinction between performance achieved with external support and learning that can later be demonstrated independently (Giannakos et al., 2025). This distinction is particularly relevant when interpreting multidimensional outcomes in GenAI-supported learning environments.

### 2.2 Project-based learning

Project-based learning (PjBL) has become one of the most widely adopted pedagogical approaches in product design education. Grounded in constructivist theory, PjBL emphasizes learning through sustained engagement in authentic, open-ended projects that simulate professional design practice (Brundiers and Wiek, 2013; Al-Balushi and Al-Aamri, 2014). Research has shown that PjBL positively influences multiple dimensions of student learning outcomes in product design education. Kuzmina et al. (2023) found that PjBL enhances systematic design thinking, enabling students to adopt structured approaches to addressing complex design problems. Similarly, Liu and Zhao (2021) argue that project-based design studios significantly enhance students' analytical reasoning and conceptual integration compared with lecture-based instruction.

PjBL is also associated with affective development. Authentic, practice-oriented tasks may enhance motivation and engagement (Zhang and Ma, 2023). In collaborative design contexts, sustained participation in project work has been linked to greater self-efficacy and stronger professional identification (Indarti, 2016; Kuppuswamy and Mhakure, 2020). Moreover, structured project-based interventions that encourage reflection and stakeholder awareness further reinforce students' sense of value and engagement in complex learning processes (Kuzmina et al., 2023). The evidence indicates that PjBL fosters sustained motivational and attitudinal development through relevance, collaboration, and iterative inquiry.

In the psychomotor domain, repeated engagement in design cycles enables students to develop and refine technical skills, including sketching, computer-aided design (CAD) modeling, and fabrication (Abellán-Nebot, 2020; Miska et al., 2022). Such experiences may contribute to technical readiness aligned with the collaborative and adaptive demands of professional practice (Parmentier et al., 2021).

Despite these reported benefits, the implementation of project-based learning in design education remains constrained by persistent structural and instructional challenges that limit its pedagogical effectiveness. The inherent complexity and open-ended nature of PjBL create ambiguous learning pathways that often increase cognitive load and overwhelm students in the absence of timely scaffolding (Murtazin et al., 2025). At the same time, instructors often struggle to provide sustained, individualized feedback to multiple project groups, particularly during critical stages such as concept development and iterative evaluation, thereby limiting opportunities for deeper learning and refinement (Sukacke et al., 2022). Collaborative project structures also reflect professional practice but may give rise to unequal participation, coordination difficulties, and performance-related stress when task allocation and assessment mechanisms are poorly designed (Schaddelee and McConnell, 2018; Chen and Yang, 2019). In addition, constraints on instructional time and rigid assessment requirements often prevent the completion of iterative cycles, resulting in fragmented learning and superficial outcomes, a challenge that is particularly pronounced among novice learners (Ali et al., 2024).

Accordingly, the effectiveness of PjBL depends on adaptive and responsive support mechanisms. A key challenge in design education research is maintaining student-centered inquiry while providing structured support for iterative processes and diverse learning demands.

### 2.3 Generative artificial intelligence as external scaffolding in learning

Generative artificial intelligence (GenAI) refers to AI systems trained on large-scale datasets that are capable of generating new content, including text, images, videos and code (Farrelly and Baker, 2023; Moorhouse et al., 2023). In design education, GenAI is commonly used in the form of large language models and text-to-image tools, enabling human-AI collaboration through prompt-based interaction (Lim et al., 2023).

The educational role of GenAI can be further understood through the concept of scaffolding. Scaffolding refers to temporary support that enables learners to perform tasks that would otherwise exceed their current level of independent competence (Wood et al., 1976). In complex learning environments, cognitive scaffolds can structure a task, direct attention to relevant information, and support planning and performance of key task components (Reiser, 2004). From this perspective, GenAI may function as cognitive scaffolding when it helps learners organize information, externalize emerging ideas, compare alternatives, and obtain timely explanatory feedback (Qian, 2025; Schmitt-Fumian et al., 2025).

GenAI may also provide metacognitive scaffolding when its use prompts learners to monitor, question, verify, and revise their own reasoning. Metacognitive scaffolds are intended to support planning, monitoring, evaluation, and regulation during learning (Azevedo and Hadwin, 2005). In the present GIPLM, these functions were operationalized through iterative prompting, verification of AI-generated claims, comparison of alternative solutions, critique of generated outputs, and reflective revision (Ranade et al., 2025). Accordingly, GenAI was treated not as a substitute for students' reasoning, but as an external support intended to structure both task performance and reflective regulation across the design process.

Within the GIPLM, these two forms of scaffolding were distributed across the project workflow rather than confined to a single episode of tool use, consistent with recent work emphasizing the pedagogical integration of GenAI across learning activities rather than its use as an isolated production tool (Gao et al., 2025). Requirement analysis and research/knowledge structuring primarily provided cognitive scaffolds by organizing information, decomposing open-ended tasks, and externalizing alternatives, functions that are consistent with established accounts of cognitive scaffolding and task structuring (Reiser, 2004; Qian, 2025). Ideation, multimodal visualization, and 3D modeling combined cognitive support with iterative comparison and representation, reflecting emerging evidence that GenAI can support conceptual exploration and iterative ideation in product design learning (Huang et al., 2024; Gao et al., 2025; Liu X. et al., 2025). Product validation, critique, and reflective presentation placed greater emphasis on monitoring, verification, justification, and revision, which correspond to metacognitive regulation and reflective scaffolding processes described in prior research (Azevedo and Hadwin, 2005; Ranade et al., 2025; Schmitt-Fumian et al., 2025). This mapping provides the theoretical rationale for treating GIPLM as a process-level scaffolding intervention rather than a content-generation condition. Because the present study did not directly measure scaffold use, metacognitive regulation, or cognitive load, these functions are specified as design features of the intervention rather than tested mediating mechanisms.

Emerging research suggests that integrating GenAI into design instruction may influence ideation processes and learning experiences. For example, embedding AI-generated content into product design courses has been associated with increased idea generation and higher perceived self-efficacy (Huang et al., 2024). Liu H. et al. (2025) further found that GenAI increases students' frequency of reflection and reduces performance disparities across cognitive style groups, thereby supporting more balanced design outcomes in collaborative conceptual design settings.

Moreover, the immediacy of AI-generated responses creates a relatively low-threat, nonjudgmental environment for exploration. Unlike traditional feedback from peers or instructors, GenAI-generated feedback does not involve explicit social evaluation, which may reduce expression anxiety and fear of failure during the early stages of ideation (Nguyen and Nguyen, 2025; Susoy, 2026). In addition, GenAI facilitates rapid visualization and multi-version prototyping, enhancing expressive fluency, iterative efficiency, and the externalization of design concepts (Gao et al., 2025; Yang and Shin, 2025). Current research suggests that GenAI in design education not only promotes cognitive gains but also enhances the quality of the learning process by reducing social evaluative pressure, strengthening reflective processes, and supporting skill development.

However, the educational value of GenAI ultimately depends on the instructional context, including task structure, assessment design, and pedagogical guidance. Fleischmann (2025) argues that when GenAI is not systematically integrated into project workflows, students tend to use it as a rapid production tool for generating preliminary visuals rather than as a scaffold for problem framing, needs analysis, and argumentative reasoning, which may weaken the depth and coherence of their design thinking. Furthermore, without structured reflection and assessment, students may become overdependent on AI-generated outputs, thereby reducing originality and compromising design agency (Bartlett and Camba, 2024). These findings do not suggest that simply introducing GenAI will improve learning outcomes. Rather, its educational effects depend on how it is integrated into instructional design and how it interacts with the cognitive, affective, and psychomotor dimensions of learning.

## 3 Methods

### 3.1 Research design

This study employed a quasi-experimental pre-test/post-test control group design to examine whether GenAI-supported PjBL is associated with differential changes in learning outcomes among undergraduate product design students. Random assignment was not feasible because the classes had been administratively scheduled before the study began. As a result, participants were assigned to either an experimental group (EG) or a control group (CG) based on their existing class enrollment. Before the intervention, both groups completed the same pre-test measures of multidimensional learning outcomes. Following the intervention, the EG completed the same PjBL project with structured GenAI support, whereas the CG completed the project using traditional PjBL supported by instructor and peer feedback. Both groups then completed the post-test under comparable conditions. Given the non-randomized pre-test–post-test design, the primary analysis employed a two-way mixed-design ANOVA, with time (pre-test vs. post-test) treated as the within-subjects factor and instructional condition (EG vs. CG) as the between-subjects factor. The Time × Group interaction was examined to determine whether changes in learning outcomes differed between the two instructional conditions. When a significant interaction was detected, follow-up simple effects analyses and pairwise comparisons were conducted to examine within-group changes and between-group differences at each time point. For advanced design application (ADA), which showed a significant baseline group difference, an analysis of covariance (ANCOVA) was additionally conducted with ADA post-test score as the dependent variable, instructional condition as the fixed factor, and ADA pre-test score as the covariate. The homogeneity-of-regression-slopes assumption was examined before interpreting the adjusted group effect.

### 3.2 Sample

During the Fall 2025 semester, sixty undergraduate students (*N* = 60) majoring in product design at a higher education institution in southeastern China participated in this study. All participants were enrolled in two parallel sections of the same course, ensuring comparable curricular and institutional conditions. A quasi-experimental design using intact groups was employed. One section served as the experimental group (*n* = 30; M age = 20.30 years, SD = 0.47) and received structured GenAI-supported PjBL, whereas the other served as the control group (*n* = 30; M age = 20.17 years, SD = 0.46) and engaged in traditional PjBL.

Because intact classes were used, the achievable sample size was limited by course enrollment. Consistent with recent methodological recommendations, a sensitivity power analysis was performed for the focal Time × Group interaction rather than based on a universal sample-size rule. The analysis assumed a 2 × 2 mixed-design ANOVA with two groups, two measurement occasions, an alpha level of 0.05, and a medium interaction effect (*f* = 0.25). With a total sample of 60 participants, the estimated statistical power was approximately 0.77 under the conservative assumption of zero correlation between repeated measures and increased to approximately 0.97 with a moderate correlation of 0.50. Accordingly, the available sample was considered adequate to detect medium or larger Time × Group interaction effects, although smaller effects may have been underpowered (Kang, 2021; Lakens and Caldwell, 2021; Lakens, 2022; Langenberg et al., 2023).

### 3.3 Instructional intervention

The quasi-experimental intervention was implemented in an undergraduate course titled *Product Improvement Design* over a 4-week period (Figure 1). Scheduled instructional time, project milestones, and formal critique opportunities were held constant across the two sections. The conditions differed in the type and availability of instructional support. The EG had access to GenAI-supported analysis, generation, visualization, and critique, whereas the conventional section relied on manual tools together with instructor and peer feedback. Because these forms of support differ in immediacy, representational affordances, and potential feedback frequency, the intervention is best understood as a bundled GenAI-supported instructional condition rather than as an isolated manipulation of AI access.

**Figure 1:**
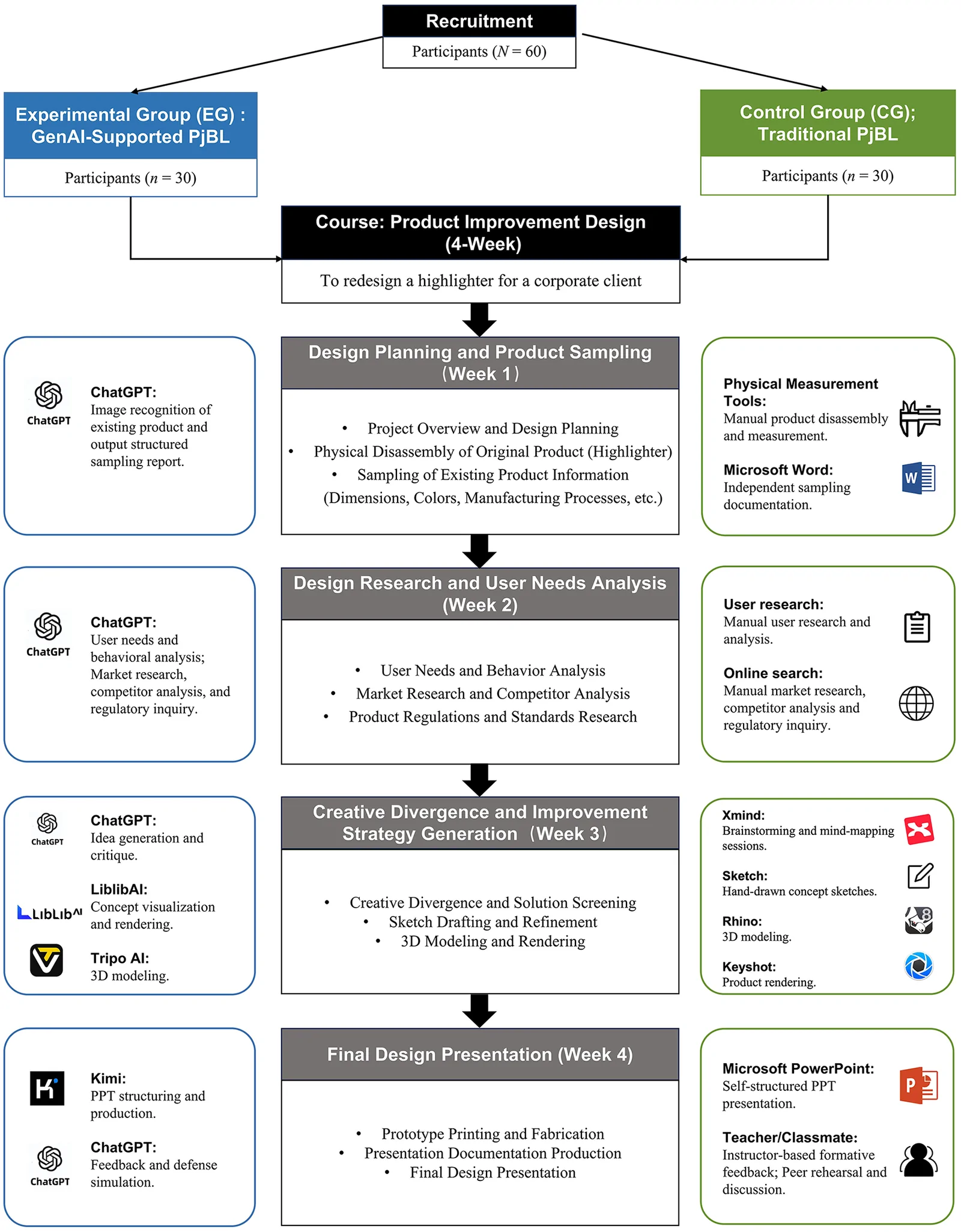
Study design and instructional activities across the two course sections.

#### 3.3.1 Experimental group: GenAI-supported PjBL

Students in the EG followed a pedagogically designed GenAI workflow embedded throughout all project stages (Figure 2). During the intervention, students used GPT-5 of ChatGPT, 2.0 version of LiblibAI, 2.5 version of Tripo AI, and K2 version of Kimi between October and November 2025. Before the project, students received instructional guidance on prompting, verification, and revision. They were encouraged to treat generated outputs as provisional materials and to evaluate them against project evidence and design constraints. Prompt histories and interaction frequencies were not systematically retained; therefore, individual variation in GenAI exposure could not be quantified. Although all students received the same stage-specific guidance and project requirements, the study could not verify whether every student implemented the intended GenAI-supported workflow with comparable frequency, depth, or consistency. The intervention should therefore be interpreted as assignment to a structured GenAI-supported condition rather than exposure to a standardized dose of GenAI use; group-level estimates may conceal meaningful within-group heterogeneity in actual use. The toolset integrated text-generation models (e.g., ChatGPT), image-generation platforms (e.g., LiblibAI), and model-generation tools (e.g., Tripo AI). These tools supported investigation, ideation, visualization, prototyping, and reflective articulation. To reduce the risk of unreflective automation, students were guided to treat GenAI outputs as provisional drafts and iteratively improve them through prompt revision and critical evaluation rather than adopting the outputs directly. This design principle aligns with recent education research emphasizing that meaningful learning occurs when students engage in iterative prompt-output dialogues and subsequently modify and refine AI-generated concepts through their own design thinking (Georgieva and Georgiev, 2025; Ranade et al., 2025). The 4-week workflow unfolded as follows:

**Figure 2:**
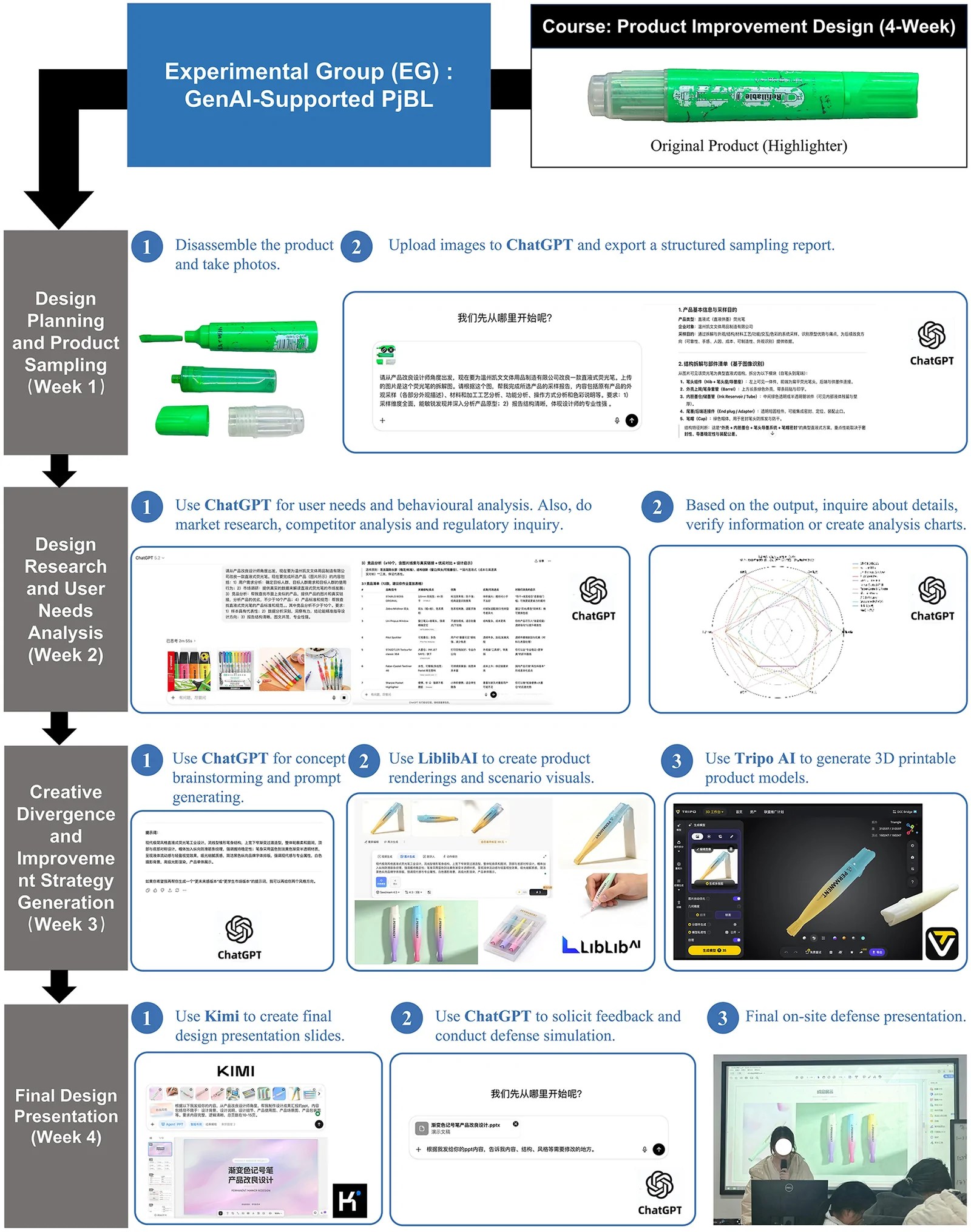
Sequence of instructional activities and GenAI-supported scaffolds used in the experimental section.

(1) AI-augmented product analysis: during product disassembly and documentation, students combined physical inspection with AI-supported structured analysis. Photographs and descriptive inputs were uploaded to ChatGPT to generate systematic analyses of form, function, materials, and ergonomic features. Students then engaged in verification loops by prompting the system to justify assumptions, provide alternative interpretations, and clarify uncertainties. This process positioned GenAI as a structured analytical scaffold while preserving empirical validation through direct physical observation.
(2) AI-supported research and knowledge structuring: GenAI was integrated as a structured research and reasoning scaffold. Students used large language models (e.g., ChatGPT) to identify target users, usage contexts, pain points, and behavioral patterns, generating structured need-behavior narratives and preliminary requirement statements. The same tools supported rapid competitor mapping through feature comparisons, positioning analysis, and the identification of differentiation signals. Students also used these tools to summarize relevant safety standards and regulatory constraints, followed by clarification of their applicable scope and subsequent verification. To avoid uncritical reliance on AI-generated outputs, learners engaged in iterative inquiry cycles by challenging assumptions, identifying missing variables, and transforming textual outputs into visual analytical artifacts (e.g., requirement hierarchies, persona journeys, and competitor matrices). These activities were intended to support evidence organization, verification, and structured design reasoning.
(3) Multi-modal generative prototyping: GenAI functioned as a multi-modal prototyping scaffold across text, image, and 3D generation. Students first employed large language models (e.g., ChatGPT) to generate multiple improvement directions and translate them into structured prompts, iteratively refining the wording to enhance both novelty and feasibility. These prompts were then applied through text-to-image platforms (e.g., LiblibAI) to produce diverse product renderings and scenario visualizations. Visual outputs were treated as exploratory hypotheses rather than finalized solutions; students documented prompt development and selected alternatives based on explicit criteria, including user alignment, manufacturability, and brand coherence. Selected concepts were further converted into preliminary 3D geometries using AI-based modeling tools (e.g., Tripo AI), enabling rapid form evaluation and prototyping preparation. Mesh refinement and dimensional adjustments were conducted to ensure ergonomic and functional plausibility. From an instructional perspective, emphasis was placed on iterative comparison, critique, and refinement rather than one-step generation.
(4) AI-mediated feedback and reflective articulation: in the final phase, GenAI supported presentation development and reflective refinement. Students used Kimi to structure and develop a coherent presentation narrative that connected problem framing, research evidence, concept evolution, and final proposals. Large language models (e.g., ChatGPT) were then employed for AI-mediated critique, providing formative feedback on conceptual clarity, evidentiary sufficiency, and potential limitations. Through simulated defense sessions, students prompted the models to assume the roles of critical reviewers, generating counterarguments and posing challenging questions. The rehearsal activity was intended to provide opportunities for students to refine their arguments and prepare for the final presentation.

#### 3.3.2 Control group: traditional PjBL

The CG completed the same 4-week redesign project using conventional project-based learning. The CG followed the same project milestones (Weeks 1–4) and produced comparable deliverables, including sampling documentation, research reports, concept sketches/prototypes, and final presentations. However, the CG relied on manual measurement and disassembly, manual user, market, and regulatory research through online searches and document review, as well as traditional ideation and modeling methods (e.g., brainstorming, mind mapping, hand sketches, and manual 3D modeling and rendering), with support from instructor and peer feedback. This condition represents a robust and conventional PjBL approach, providing a clear basis for comparison with the structured GenAI-supported workflow.

### 3.4 Instruments

The Product Design Learning Outcomes Questionnaire (PDLOQ) was developed to assess multidimensional learning outcomes in undergraduate product design. The instrument is theoretically grounded in Bloom's Taxonomy and its subsequent revisions and extensions (Anderson and Krathwohl, 2001; Dave, 1970; Krathwohl et al., 1964). The 18-item instrument includes six three-item subscales: cognitive understanding (CU), problem-solving and creativity (PSC), engagement and participation (EP), ethical and user-centered responsibility (EUCR), fundamental tool operation (FTO), and advanced design application (ADA). CU and PSC were mapped onto cognitive learning outcomes, EP and EUCR onto affective outcomes, and FTO and ADA onto self-perceived competence in psychomotor-related design activities. All items were rated on a 7-point scale ranging from 1 (strongly disagree) to 7 (strongly agree). Accordingly, FTO and ADA should be interpreted as students' self-perceived competence in design-related technical and application activities rather than as direct measures of independently demonstrated psychomotor performance.

The preliminary psychometric properties of the PDLOQ were examined in a pilot study involving 103 undergraduate product design students. Exploratory factor analysis (EFA) identified the proposed dimensions of the pilot instrument, with item loadings for the six learning-outcome subscales ranging from 0.781 to 0.911 and no substantial cross-loadings. These results provided preliminary support for the intended multidimensional structure of the PDLOQ. Internal consistency was also satisfactory, with Cronbach's alpha values of 0.943 for CU, 0.933 for PSC, 0.907 for EP, 0.911 for EUCR, 0.911 for FTO, and 0.866 for ADA.

In addition to self-reported learning outcomes, students' academic performance was included as an independently assessed performance indicator of instructional effectiveness. Specifically, midterm grades (MG) and final grades (FG) were collected from both the experimental and control groups and treated as repeated measures to examine changes in performance across the instructional cycle. Course grades were derived from standardized assessment components aligned with the course syllabus. The midterm grade reflected students' progress during the initial project phase, including design research, problem framing, and preliminary ideation. Final grades were evaluated using structured rubrics that assessed novelty, feasibility, and overall design quality. To minimize potential bias, the same evaluation criteria were applied to both groups, and the grading procedures remained consistent throughout the semester. Importantly, final project submissions were anonymized prior to assessment, and evaluators were not informed of students' group assignments during the grading process. This procedure was implemented to reduce expectancy effects and strengthen the internal validity of performance comparisons.

### 3.5 Data collection

Data were gathered across a 4-week instructional cycle in an undergraduate “Product Improvement Design” course. Two intact classes participated in the study, with one assigned to the GenAI-supported PjBL condition and the other to the traditional PjBL framework. To secure measurement accessibility and linguistic equivalence, the PDLOQ items underwent translation into Chinese and back-translation into English, strictly adhering to established cross-language instrument adaptation protocols (Brislin, 1970). Discrepancies between the original and back-translated versions were resolved through iterative reviews by bilingual researchers and course instructors to achieve semantic clarity and contextual fit for product design learning.

A two-wave survey design was employed for data collection. At the start of Week 1, students completed the PDLOQ pre-test through an online survey platform. Upon project completion in Week 4, the same instrument was re-administered as the post-test under identical conditions. Survey responses were numerically coded and converted into subscale scores. Beyond these self-reported outcomes, objective academic performance data were retrieved directly from official course records. Specifically, midterm and final grades were collected for both groups to serve as repeated achievement indicators across the instructional cycle. All datasets were anonymized using numerical identifiers prior to analysis to maintain confidentiality.

### 3.6 Data analysis

Data were analyzed using IBM SPSS Statistics 27. Prior to inferential analyses, the data were screened for missing values, outliers, normality, and homogeneity of variance. Descriptive statistics were calculated for each outcome variable by group and time point. To examine whether learning outcomes changed differently across instructional conditions, mixed-design ANOVAs were conducted, with time as the within-subjects factor and instructional condition as the between-subjects factor. The Time × Group interaction was treated as the primary test of differential change associated with the instructional condition. Partial eta squared was reported as the effect size for ANOVA results. Given the number of outcome variables, the same mixed-design ANOVA framework was applied to the academic performance indicators, including midterm and final grades. Because the study involved intact classes rather than random assignment, all results were interpreted cautiously as evidence of differential developmental patterns rather than definitive causal effects. Because ADA showed a significant baseline group difference, an additional one-way ANCOVA was conducted for this outcome, with ADA post-test scores as the dependent variable, instructional condition as the fixed factor, and ADA pre-test scores as the covariate. Before interpreting the ANCOVA, homogeneity of regression slopes was evaluated using the Group × ADA pre-test interaction. Partial eta squared was reported as the effect size for the ANCOVA.

## 4 Results

### 4.1 Preliminary assumption test

Before primary analyses, the data were screened to verify compliance with parametric test assumptions. Kolmogorov-Smirnov tests indicated no significant departures from normality across the six pre-test PDLOQ subscales (*p*-values ranging from 0.053 to 0.200). Levene's tests similarly confirmed equal variances for each pre-test subscale. These findings validated the application of mixed-design ANOVA. Because the within-subjects factor comprised only two levels, Mauchly's test of sphericity was not required. For the ADA ANCOVA, the Group × ADA pre-test interaction was not significant, *F*_(1, 56)_ = 1.292, *p* = 0.261, partial η^2^ = 0.023, supporting the homogeneity-of-regression-slopes assumption. Levene's test for the ANCOVA model was also non-significant, *F*_(1, 58)_ = 0.004, *p* = 0.952, supporting homogeneity of error variance.

### 4.2 Baseline equivalence

At baseline, experimental and control groups showed no significant differences in CU, PSC, EP, EUCR, or FTO. A significant pre-test disparity emerged solely for ADA, with the control group scoring higher than the experimental group. Given this initial imbalance, ADA outcomes were interpreted cautiously, relying primarily on the Time × Group interaction to evaluate differential change trajectories over time. Given this initial imbalance, an additional ANCOVA was conducted to determine whether the post-test group difference in ADA remained significant after controlling for baseline ADA scores.

### 4.3 Mixed-design ANOVA for multidimensional learning outcomes

Table 1 summarizes the changes in learning outcomes across the two instructional conditions. Overall, both experimental and control groups exhibited pre-to-post-test gains across all six PDLOQ dimensions. However, the magnitude of improvement varied by domain and instructional condition.

**Table 1:** Changes in learning outcomes by instructional condition from pre-test to post-test.

Outcome	Group	Pre-test mean ±SD	Post-test mean ±SD	Within-group		Between-group		Time × Group *F*(*p*)	Partial η^2^
				Change mean difference	*p*	Pre-test *F*(*p*)	Post-test *F*(*p*)		
CU	Control group	3.744 ± 0.762	4.256 ± 0.781	0.512 [0.117, 0.906]	0.012	3.608 (0.062)	21.887 (^***^)	4.302 (0.043)	0.069
	Experimental group	4.189 ± 1.031	5.278 ± 0.906	1.089 [0.695, 1.483]	^***^				
PSC	Control group	4.122 ± 0.864	4.567 ± 0.935	0.445 [−0.049, 0.939]	0.077	3.183 (0.080)	8.186 (0.006)	10.132 (0.002)	0.149
	Experimental group	3.712 ± 0.917	5.267 ± 0.961	1.555 [1.061, 2.049]	^***^				
EP	Control group	4.844 ± 0.978	5.178 ± 0.762	0.333 [−0.060, 0.727]	0.095	1.137 (0.291)	3.029 (0.087)	0.193 (0.662)	0.003
	Experimental group	5.133 ± 1.116	5.589 ± 1.046	0.456 [0.062, 0.849]	0.024				
EUCR	Control group	5.055 ± 0.822	5.489 ± 0.741	0.433 [0.072, 0.795]	0.020	1.873 (0.096)	0.176 (0.084)	0.092 (0.762)	0.002
	Experimental group	5.367 ± 0.936	5.878 ± 0.957	0.511 [0.149, 0.873]	0.006				
FTO	Control group	4.389 ± 1.188	4.555 ± 0.808	0.166 [−0.380, 0.712]	0.545	0.245 (0.622)	15.514 (^***^)	7.343 (0.009)	0.112
	Experimental group	4.245 ± 1.061	5.456 ± 0.957	1.211 [0.665, 1.756]	^***^				
ADA	Control group	4.078 ± 1.147	4.299 ± 0.774	0.221 [−0.222, 0.664]	0.322	5.451 (0.023)	18.333 (^***^)	23.703 (^***^)	0.290
	Experimental group	3.434 ± 0.984	5.178 ± 0.816	1.744 [1.301, 2.187]	^***^				

Values are presented as mean ± standard deviation unless otherwise indicated. Within-group changes were estimated as post-test minus pre-test scores. Between-group difference in change represents the experimental group change minus the control group change. Time × Group p-values were obtained from mixed-design ANOVA. ^***^p < 0.001.

For the cognitive domain, significant Time × Group interaction effects emerged for both CU and PSC. Regarding CU, the control group score increased from 3.744 ± 0.762 to 4.256 ± 0.781, whereas the experimental group increased from 4.189 ± 1.031 to 5.278 ± 0.906. While within-group gains were significant for both conditions, the experimental group showed a pronounced increase, yielding a significant Time × Group interaction, *F* = 4.302, *p* = 0.043, η^2^ = 0.069. Furthermore, the post-test between-group difference was significant, *F* = 21.887, *p* < 0.001, indicating that the experimental group had significantly higher post-test CU scores than control group. For PSC, the control group displayed a non-significant increase [MD = 0.445, 95% CI (−0.049, 0.939), *p* = 0.077], whereas the experimental group showed a marked increase [MD = 1.555, 95% CI (1.061, 2.049), *p* < 0.001]. The Time × Group interaction reached significance, *F* = 10.132, *p* = 0.002, η^2^ = 0.149, indicating that GenAI-supported PjBL was associated with greater gains in problem-solving and creativity than traditional PjBL.

For the affective domain, both EP and EUCR increased over time; however, Time × Group interaction effects failed to reach significance. For EP, the control group scores rose from 4.844 ± 0.978 to 5.178 ± 0.762, while experimental group scores increased from 5.133 ± 1.116 to 5.589 ± 1.046. Although the experimental group exhibited significant within-group improvement [MD = 0.456, 95% CI (0.062, 0.849), *p* = 0.024], the Time × Group interaction was non-significant (*F* = 0.193, *p* = 0.662, η^2^ = 0.003). Similarly, for EUCR, both the control group [MD = 0.433, 95% CI (0.072, 0.795), *p* = 0.020] and the experimental group [MD = 0.511, 95% CI (0.149, 0.873), *p* = 0.006] showed significant within-group gains. Nonetheless, the Time × Group interaction remained non-significant (*F* = 0.092, *p* = 0.762, η^2^ = 0.002). These findings indicate that while affective outcomes improved across the intervention, GenAI-supported PjBL yielded no superior gains over traditional PjBL.

For the psychomotor domain, significant Time × Group interaction effects were observed for both FTO and ADA. For FTO, the control group demonstrated a non-significant increase from 4.389 ± 1.188 to 4.555 ± 0.808 [MD = 0.166, 95% CI (−0.380, 0.712), *p* = 0.545]. In contrast, the experimental group showed a substantial increase from 4.245 ± 1.061 to 5.456 ± 0.957 [MD = 1.211, 95% CI (0.665, 1.756), *p* < 0.001]. The Time × Group interaction was significant, *F* = 7.343, *p* = 0.009, η^2^ = 0.112, indicating that GenAI-supported PjBL was associated with greater gains in students' fundamental tool operation skills. For ADA, the control group exhibited a non-significant increase [MD = 0.221, 95% CI (−0.222, 0.664), *p* = 0.322], whereas the experimental group demonstrated a marked improvement [MD = 1.744, 95% CI (1.301, 2.187), *p* < 0.001]. The Time × Group interaction was significant with a relatively large effect size, *F* = 23.703, *p* < 0.001, η^2^ = 0.290. As shown in Table 2, after controlling for pre-test ADA scores, the effect of instructional group on post-test ADA remained significant, *F*_(1, 57)_ = 20.519, *p* < 0.001, partial η^2^ = 0.265. In contrast, the ADA pre-test covariate was not statistically significant, *F*_(1, 57)_ = 1.903, *p* = 0.173, partial η^2^ = 0.032. These results indicate that the higher post-test ADA scores in the experimental group cannot be explained solely by the initial baseline difference between the two groups.

**Table 2:** ANCOVA for ADA post-test scores controlling for pre-test ADA.

Source	Type III sum of squares	df	Mean square	*F*	*p*	Partial Eta squared
Group	12.779	1	12.779	20.519	< 0.001	0.265
ADA pre-test	1.185	1	1.185	1.903	0.173	0.032
Error	35.499	57	0.623	—	—	—

ADA post-test was the dependent variable, group was the fixed factor, and ADA pre-test was the covariate. Assumption checks for the ANCOVA are reported in Section 4.1.

The results indicate that patterns of change differed across learning domains under the two instructional conditions. Significantly larger between-group gains were observed in the cognitive domain, particularly in CU and PSC, as well as in the psychomotor domain, particularly in FTO and ADA. In contrast, affective outcomes, including EP and EUCR, improved over time in both groups but did not differ significantly between the two instructional conditions. GenAI-supported condition was associated with larger gains in cognitive processing, creative problem-solving, tool operation, and advanced design application, whereas no significant differential gains were observed for affective outcomes.

### 4.4 Differential developmental trajectories across domains

Visual inspection of the pre-post trajectories indicates that GenAI-supported PjBL was associated with different developmental trajectories, with the magnitude of this influence varying across domains (Figure 3). In the cognitive domain, the experimental group exhibited a steeper positive slope than the control group in both cognitive understanding and problem-solving and creativity. Notably, PSC demonstrated a clear divergence pattern: although both groups improved over time, the experimental group showed substantially greater growth, resulting in a pronounced widening of the between-group gap at post-test.

**Figure 3:**
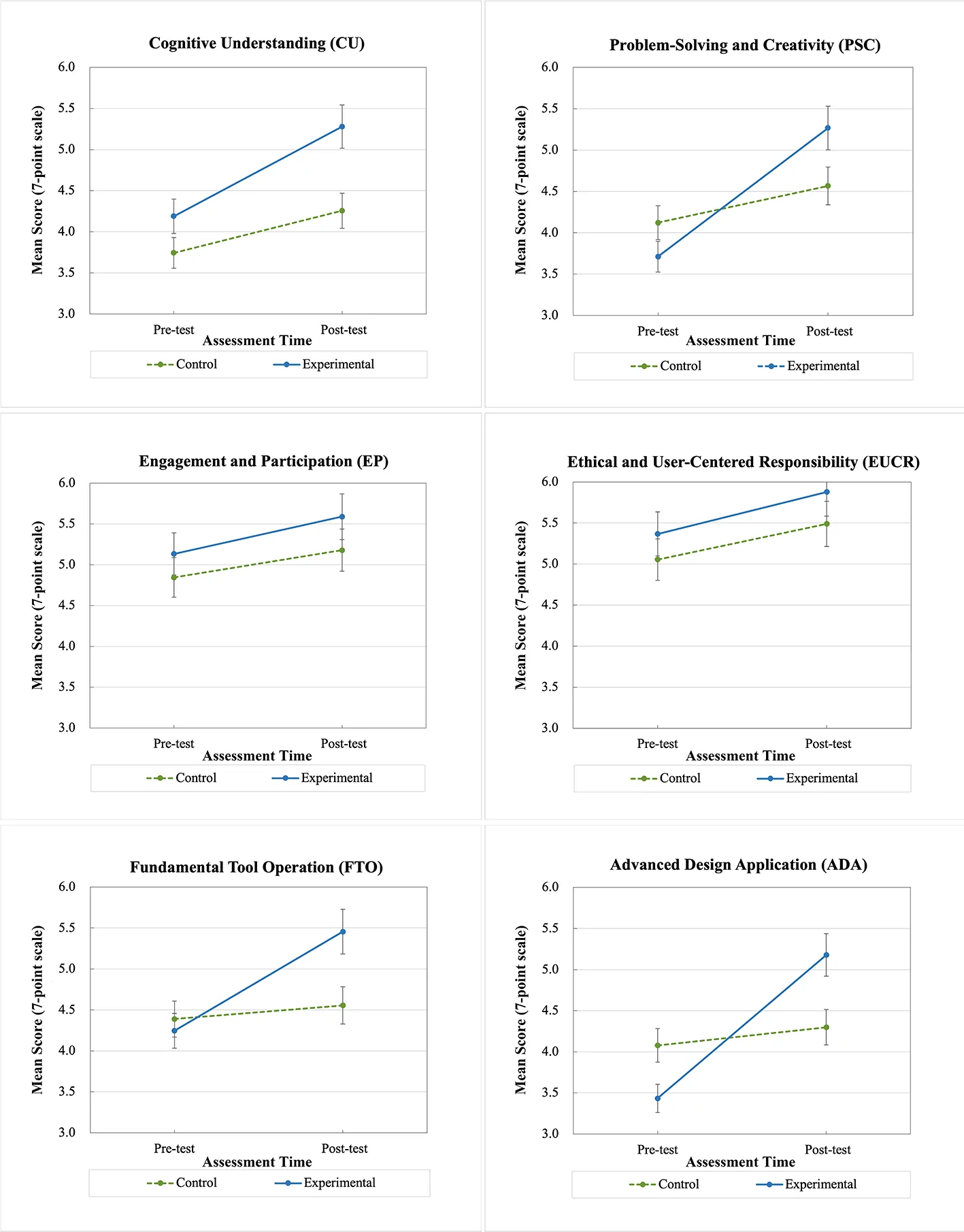
Developmental trajectories of learning outcomes across domains (pre-test vs. post-test).

The between-group divergence was more pronounced in the psychomotor-related domain. For fundamental tool operation and advanced design application, the experimental group demonstrated substantially greater growth from pre-test to post-test compared with the control group. In particular, the ADA trajectory exhibited a clear pattern of convergence and advancement: despite starting from a lower pre-test score, the experimental group achieved a higher post-test mean than the control group, indicating a substantial difference over time.

By contrast, the affective domain exhibited more parallel developmental patterns. Both groups showed modest increases in engagement and participation and ethical and user-centered responsibility, with limited widening of group differences at post-test. This suggests that the two conditions showed similar affective gains, whereas larger between-group differences were observed in cognitive and applied-performance outcomes.

### 4.5 Academic performance

Although the PDLOQ captured students' self-reported perceptions of multidimensional learning outcomes, it did not directly assess independently evaluated academic performance. Therefore, midterm grades (MG) and final grades (FG) were analyzed as complementary performance-based indicators, focusing on within-group changes over time and between-group differences. These course-grade analyses are therefore interpreted as complementary evidence of assessed project performance, not as a direct validation of PDLOQ self-perceptions or as evidence of the mechanism linking perceived competence to performance.

Descriptive statistics for midterm and final course grades are presented in Table 3. At midterm, the experimental (*M* = 77.967, SD = 6.478) and control groups (*M* = 77.583, SD = 9.013) showed highly comparable mean scores. At final assessment, the experimental group achieved a substantially higher mean score (*M* = 87.000, SD = 4.624) than the control group (*M* = 77.983, SD = 8.703). The overall mean increased from 77.775 (SD = 7.784) at midterm to 82.492 (SD = 8.271) at final assessment.

**Table 3:** Descriptive statistics of the course grade.

Assessment	Groups	Mean	SD	*N*
MG	Control group	77.5833	9.01252	30
	Experimental group	77.9667	6.47799	30
	Total	77.7750	7.78385	60
FG	Control group	77.9833	8.70343	30
	Experimental group	87.0000	4.62378	30
	Total	82.4917	8.27109	60

Note: MG, midterm grade; FG, final grade.

To examine changes in course grades over time and differences between instructional conditions, a mixed-design ANOVA was conducted with time (MG vs. FG) as the within-subject factor and group as the between-subject factor. As shown in Table 4, the main effect of time was significant, *F*_(1, 58)_ = 61.878, *p* < 0.001, partial η^2^ = 0.516, indicating that significant grade changes from midterm to final assessment. More importantly, the Time × Group interaction was also significant, *F*_(1, 58)_ = 51.828, *p* < 0.001, partial η^2^ = 0.472, demonstrating substantially different grade changes between the experimental and control groups.

**Table 4:** Tests of within-subjects effects for the course grade (Greenhouse-Geisser).

Source	Type III sum of squares	df	Mean square	*F*	*p*	Partial Eta squared
Time	667.408	1.000	667.408	61.878	0.000	0.516
Time ^*^ groups	559.008	1.000	559.008	51.828	0.000	0.472
Error (time)	625.583	58.000	10.786			

^*^ The mean difference is significant at the .05 level.

Pairwise comparisons were conducted to clarify the interaction effect and are presented in Table 5. The control group showed a small, non-significant increase from midterm to final assessment [MD = 0.400, SE = 0.848, *p* = 0.639, 95% CI (−1.297, 2.097)]. In contrast, the experimental group demonstrated a substantial, significant increase [MD = 9.033, SE = 0.848, *p* < 0.001, 95% CI (7.336, 10.731)]. Between-group comparisons showed no significant difference at midterm, *F*_(1, 58)_ = 0.036, *p* = 0.851, confirming initial comparability in academic performance. However, a significant group difference emerged at final assessment, *F*_(1, 58)_ = 25.111, *p* < 0.001, with the experimental group achieving higher final grades than the control group.

**Table 5:** Pairwise comparisons of course grades.

Groups	Tests		Mean difference (I–J)	SE	*p*	Lower bound	Upper bound
Control group	Pre-test (J)	post-test (I)	0.400	0.848	0.639	−1.297	2.097
Experimental Group	Pre-test (J)	post-test (I)	9.033^*^	0.848	0.000	7.336	10.731
*F*	0.036	25.111					
*p*	0.851	0.000					

Mean differences represent within-group comparisons between pre-test and post-test. The *p*-values reported at the bottom of the table indicate between-group differences at each testing phase. ^*^ The mean difference is significant at the .05 level.

Overall, the results indicate that the experimental and control groups began with comparable academic performance but followed markedly different developmental patterns thereafter. While the control group showed minimal change, the experimental group achieved substantial improvements in final course grades. These results indicate that GenAI-supported PjBL was associated with greater gains in academic performance in addition to the domain-specific learning outcomes assessed by the PDLOQ.

## 5 Discussion

### 5.1 GenAI-supported PjBL as a process-level intervention in product design education

The clearest pattern in the results is that the largest between-group differences appeared in activities where students had to move repeatedly between information, representations, and design decisions. Larger pre-to-post increases were observed for CU, PSC, FTO, ADA, and final course performance in the GenAI-supported section, while the intervention itself required students to use GenAI across analysis, research, ideation, visualization, modeling, critique, and presentation. A workflow-level interpretation is therefore plausible. Recent design-education research also points toward the importance of how GenAI is embedded in teaching. Fleischmann (2024) argues for more formal integration of GenAI into design curricula because students often use these tools in *ad hoc* ways, and Schmitt-Fumian et al. (2025) found that AI-generated feedback can support iterative idea refinement. Gao et al. (2025) likewise show that GenAI is being used across several stages of product design rather than only at the point of final output. Iranmanesh (2026) frames this developing relationship as reflective co-action within studio pedagogy. The present findings are compatible with this literature, although the study did not isolate the contribution of any single stage of the GIPLM.

The results also need to be read alongside evidence of possible costs. Wadinambiarachchi et al. (2024) found that exposure to AI-generated examples can increase design fixation and reduce the variety and originality of ideas. Bartlett and Camba (2024) raise related concerns about originality, authorship, and the reliability of visually persuasive outputs in product design education. Fleischmann (2025) further notes that rapid production can pull students toward output generation when pedagogical framing is weak. The GIPLM attempted to limit these risks by treating generated material as provisional. Students were asked to verify claims, compare alternatives, revise prompts, and retain responsibility for final design decisions. The observed pattern is compatible with a workflow-level interpretation, although the present data do not establish this as a mechanism. Prompt histories and detailed decision traces were not systematically retained. This matters because Wadinambiarachchi et al. (2024) also suggest that prompt strategy and the way learners respond to AI suggestions can shape co-ideation. The observed group differences should therefore be interpreted as associated with the bundled instructional condition. The present data do not estimate a dose-response effect for GenAI use.

### 5.2 Domain-sensitive patterns from an educational psychology perspective

The cognitive results are strongest for PSC and are also evident for CU. One possible account is that GenAI shortened some of the work required to externalize and compare intermediate ideas, which may have left more opportunities within the project schedule for evaluation and revision. Huang et al. (2024), in product design ideation teaching, reported gains in creative ideation and self-efficacy with artificial intelligence generated content (AIGC). Schmitt-Fumian et al. (2025) similarly found that rapid AI feedback supported iterative refinement during brainstorming. Evidence from broader higher-education research suggests that this relationship is not straightforward. Fan et al. (2025) showed that AI-supported learners can display different self-regulated learning processes and performance patterns without equivalent gains in motivation or knowledge transfer. The present data fit this conditional picture. CU and PSC improved more in the experimental group, but cognitive load, metacognitive monitoring, reasoning sequences, and time-on-task allocation were not measured. The results therefore support stronger cognitive outcomes under the GIPLM condition. Claims about reduced cognitive load or altered metacognitive regulation remain provisional.

The affective pattern is different. EP and EUCR increased in both groups, yet the Time × Group interactions were very small and non-significant. This finding should be read alongside a mixed body of evidence. Chen et al. (2025) reported higher motivation and self-efficacy in a design and art course using a ChatGPT-driven pedagogical agent, while Hwang and Wu (2025) linked GenAI use with self-efficacy and anxiety in design students. Lu et al. (2025) also found generally positive attitudes toward AI-assisted product design. The present intervention, however, gave both groups the same authentic project, project milestones, critique activities, collaboration, and final presentation requirements. Some affective improvement may therefore have come from the shared PjBL experience. EP and EUCR also began at relatively high levels on the seven-point scale, leaving less room for separation over 4 weeks. Fan et al. (2025) is relevant here because their experiment found differences in learning processes and performance across support conditions without a corresponding difference in post-task intrinsic motivation. A similar separation between performance-related change and short-term affect may have occurred in this study. From a cognitive load perspective, learning to formulate prompts and judge AI output could also create temporary adaptation demands (Sweller, 1988). Since neither cognitive load nor adaptation effort was measured, that explanation remains tentative.

FTO and ADA require a more careful interpretation because both are self-reported. They capture students' perceived competence in tool operation and advanced design application, rather than independently demonstrated psychomotor mastery. Even with that limitation, the ADA result is comparatively robust. After adjustment for the baseline difference, the post-test group effect remained significant, *F*_(1, 57)_ = 20.519, *p* < 0.001, partial η^2^ = 0.265. One possible educational explanation is that multimodal GenAI may have reduced representational friction and may have facilitated movement between textual descriptions, visual concepts, and preliminary 3D forms. Gao et al. (2025) describe comparable uses of GenAI across ideation, prototyping, evaluation, and design development, while Huang et al. (2024) found that AIGC could support product-design ideation even though benefits varied by learner characteristics and outcome type. These studies make a procedural interpretation plausible, but they do not establish independent technical mastery in the present sample. The course-grade results provide a separate source of evidence. Midterm performance was comparable, whereas the experimental group improved markedly by the final assessment under anonymized, rubric-based grading. This pattern is consistent with product-design studies reporting improved learning outcomes under AI-supported instruction (Huang et al., 2024; Lu et al., 2025). The convergence between PDLOQ changes and final grades is useful because the two measures capture different forms of evidence. It should not be turned into a causal chain. The study did not test whether self-efficacy, perceived competence, cognitive friction, or another mediator explained the grade difference. A more defensible conclusion is that the GIPLM condition was associated with stronger changes in several perceived learning outcomes and with higher final course performance, while the processes connecting those outcomes remain unresolved.

## 6 Limitations

Several limitations should be acknowledged. Firstly, the quasi-experimental design within a single institutional context may limit generalizability. Secondly, the intervention spanned one project cycle, constraining understanding of longer-term developmental effects. Thirdly, although grading procedures were standardized, residual instructional and contextual influences cannot be entirely excluded. A further limitation concerns the absence of fine-grained process data on students' actual GenAI use. Prompt histories, interaction frequency, iteration patterns, and students' decisions to accept, reject, verify, or revise AI-generated outputs were not systematically retained. Consequently, individual variation in intervention exposure and implementation fidelity could not be quantified. The study therefore cannot distinguish whether the observed group-level gains were associated more strongly with interaction frequency, prompt quality, verification behavior, iteration depth, or selective uptake of AI suggestions. The micro-level mechanisms through which human-AI co-creation contributed to the observed outcomes remain only partially observable, and the reported group means may conceal meaningful within-group heterogeneity. Future research should employ longitudinal and cross-institutional designs to examine longer-term changes associated with GenAI-supported learning. Process-tracing methods, such as learning analytics and interaction log analysis, may provide finer-grained evidence of how GenAI tools relates to iteration patterns and cognitive effort allocation (Gašević et al., 2015). Additionally, further investigation into collaborative regulation, identity formation, and ethical reasoning in AI-supported PjBL would deepen understanding of the affective boundary conditions observed in this study.

## 7 Conclusion

This study examined changes asscociated with a structured GenAI-supported PjBL module on multidimensional learning outcomes and academic performance in undergraduate product design education. The findings indicate that embedding GenAI across the project workflow was associated with greater improvements in cognitive understanding, problem-solving and creativity, self-perceived fundamental tool operation, self-perceived advanced design application, and final course performance than traditional PjBL. The ADA post-test difference remained significant after controlling for baseline ADA scores, strengthening the robustness of this specific finding. In contrast, engagement and participation, as well as ethical and user-centered responsibility, improved in both groups without significant differences between conditions, suggesting that these affective outcomes showed similar gains across both conditions, with no significant Time × Group interactions. Overall, the findings are consistent with the potential value of integrating GenAI as a structured and accountable scaffold for research, ideation, visualization, modeling, evaluation, and revision rather than as an isolated content-generation tool. Given the quasi-experimental design, modest sample size, short intervention period, and reliance on self-reported measures for several outcomes, the findings should be considered preliminary. Future research should examine longer-term effects, independent skill transfer, design process data, and the conditions under which GenAI support is associated with stronger learning without diminishing students' critical judgment and design agency.

## Data Availability

The datasets presented in this article are not readily available because the de-identified data supporting the findings of this study are available from the corresponding author upon reasonable request, subject to applicable ethical and institutional requirements. Data that could potentially compromise participant confidentiality will not be shared. Requests to access the datasets should be directed to Yizhou Chen, yzchenzhou@gmail.com.
